# Materials aesthetics: A replication and extension study of the conceptual structure

**DOI:** 10.1371/journal.pone.0277082

**Published:** 2022-11-02

**Authors:** Barbara E. Marschallek, Thomas Jacobsen

**Affiliations:** Experimental Psychology Unit, Helmut Schmidt University / University of the Federal Armed Forces Hamburg, Hamburg, Germany; Rose-Hulman Institute of Technology / Indiana University, UNITED STATES

## Abstract

Natural occurrences and the choice of specific materials have a major impact on the experience of the physical environment. The results of a recent study using a free listing task involving only adjectives suggested that the conceptual structure of the aesthetics of materials is structured by sensorial, neutrally valenced, descriptive terms, while showing no primacy of beauty. The present article examined the conceptual structure underlying the aesthetic experience of various materials using a different methodological approach. Applying a technique based upon semantic differentials, individuals in the present study (*n* = 272) were asked to judge the applicability of the most frequently listed terms in the previous study to the aesthetics of different materials. Overall, the results of multiple analyses yielded a converging picture for the two studies. Additionally, as materials constitute the basis of complete entities, the role of products in the conceptual representation of the aesthetics of materials was investigated with an exploratory approach. No support was found for the hypothesis that products play such a role. Finally, limitations regarding the outcome of the present study are discussed. All things considered, the results of this study highlight the uniqueness of the aesthetics of materials and its distinctness from the conceptual representations underlying most other aesthetic domains.

## Introduction

Individuals often judge whether entities of interest are perceived as *beautiful* or *aesthetically* positive in general. This may apply to ordinary consumer goods, such as clothing, vehicles, and furniture, as well as to natural landscapes and buildings. Because materials form the basis for a variety of these entities, their value in the overall (aesthetic) experience of the natural and built environment should be emphasized. *Materials experience*, an expression formalized by Karana et al. [1], refers to the experience that people have with, and through, a product’s material(s). It acknowledges their fundamental role in the overall product experience–and potentially applies to other entities as well (for a review of materials experience, see [2]). Identical with the framework of product experience by Desmet and Hekkert [3], Karana et al. argue that three components are ubiquitous in the experience of materials in design as well: (1) materials can differently gratify the senses, (2) evoke meanings, and (3) elicit emotions. Furthermore, Ashby and Johnson [4] have pointed out that people expect function as well as delight in the products they purchase and conclude that materials have two roles in product design: to provide technical functionality and to create product personality.

We further assume that materials themselves can even be an entity of an *aesthetic experience* [5]. Yet, the conception of aesthetics is multifold and largely depends on the scientific discipline of its use. In general, we understand *aesthetic processing* as an evaluative reception of a sensorial entity with respect to the conceptual system, primarily the beauty concept [6]. Following Fechner’s [7] “aesthetics from below” [cf. 8], the preeminence of this concept in aesthetics has been demonstrated in international and German studies, in which individuals were asked to list terms that could be used to describe the aesthetics of different domains. *Beautiful* turned out the primary term for the conceptual structures, that is, long-term memory representations, for the aesthetics of objects [9], music [10, 11], various visual stimuli [12] and visual arts [10], literature [10, 13], and tattoos [14]. But what does the conceptual structure underlying the aesthetics of materials look like? The first contact with materials is, inter alia, shaped by the interplay of (human) senses and the materials’ sensorial attributes [4, 15–17]. That is, tactile, visual, acoustic, olfactory, and gustatory features of materials positively and/or negatively affect the senses and may influence an aesthetic processing of this entity, for example, whether they are judged to be beautiful or the like. But can the primacy of the beauty dimension be assumed for the conceptual structure of this content domain?

As a potential contribution to this desideratum of research, in a recent study [18] we investigated material non-experts’ conceptual structure of the aesthetics of various materials. Drawing on a free listing task introduced by Jacobsen et al. [9] and subsequently applied in further studies [10–14, 19], we asked a final sample of 2,452 German participants to list adjectives used to describe the aesthetics of materials. Thus, the conceptual system was studied with reference to verbally available concepts. In particular, materials in general, ceramics, glass, leather, metal, paper, plastic, stone, textiles, and wood were systematically investigated, based on a classification of material substances [20]. *Smooth* was by far the most frequently listed term. In general, sensorial qualities turned out to be the central concepts, with the great majority being haptic qualities. *Beautiful* was not one of the most frequently mentioned terms. Additionally, visual analyses revealed (1) terms with cross-material significance, for instance, s*mooth*, as well as (2) material-specific characteristics, for instance, *scratchy* for textiles. Overall, the results suggested that the conceptual structure of the aesthetics of materials is diversified and rich, pointing to a primacy of sensorial, neutrally valenced, descriptive concepts. Additionally, by virtue of showing a diminished relevance of the beauty dimension, we concluded that the conceptual structure of the aesthetics of materials differs from the conceptual structures underlying other aesthetic domains.

Even though language-based methods cannot provide information about participants’ previous (material) exposure and preferences [13], they offer valuable insights into the mental representations of conceptual structures and organizations of the semantic fields of people sharing the same linguistic background [21–24]. It is argued, however, that the prevalence of the beauty concept in aesthetics varies as a function of the object class [12]. Generally, materials constitute the basis for complete products; stone, for instance, is often essential for edifices. Thus, individuals mostly encounter materials as an outcome of various production processes. Objects (used as an overarching term for entities in the physical environment) carry (social) meanings [3, 25], such as semantic associations related to lifestyles [26]. For example, specific building materials display both intrinsic and sociocultural meanings, which convey information about the social identity of homeowners [27]. Furthermore, as materials are indispensable for the physical surroundings, environmental–psychological attitudes and schemata could also be influential. Hence, the conceptual structure of the aesthetics of materials may depend on the domain of the materials’ eventual use, that is, the specific objects that incorporate them or the places they occur. Karana et al. ([28]; for an overview see [15]), for example, collected descriptive terms for the materials wood, plastics, ceramics, glass, and metal, from individuals varying in levels of design expertise. Materials were either presented as words, as samples, or as products. The authors also collected descriptions from other related sources (e.g., magazines). Interestingly, in relation to the framework of product experience [3], the collected terms could be classified into seven descriptive categories: use, manufacturing process, technical, sensorial, expressive/semantic, associative, and emotional descriptions, with the majority being expressive/semantic, that is, “qualities that a specific material expresses” in a certain product [15] (p. 47), followed by sensorial descriptions, that is, “terms making reference to interactions between materials and users through the five senses” [15] (p. 45). In our previous study [18], however, the participants were not instructed to write down adjectives that could be used to describe the aesthetics of various materials in relation to specific objects and materials were presented as words only. We cannot rule out that some participants automatically thought of specific objects or products rather than of materials per se. In this case, they might have written down object-/product-dependent associations, that is, adjectives regarding the respective material in question as incorporated in a specific object or product.

Therefore, the additional application of other (language-based) methodological approaches can help in assessing the stability of our previous results. Furthermore, as materials can be regarded a domain of interdisciplinary interest (e.g., [29]), including design, engineering, manufacturing, crafts, and psychological perspectives, alternative studies to the existing ones, for example, from the field of design, seem worthwhile. The investigation of the conceptual structure underlying the same as well as additional materials may then be approached. To this end, the present article presents a conceptual replication of our previous study using a different methodological approach. In addition, as materials constitute the basis of complete entities and individuals mostly encounter materials as an outcome of various production processes, the role of products in the conceptual structure of materials aesthetics was investigated using an exploratory approach.

As the domain of materials ought to be investigated from the point of view of several stakeholders, this replication should involve an interdisciplinary perspective utilizing a method widely acknowledged in research. Additionally, as has been shown for music reception [30], a combined bottom-up and top-down approach may be well suited for capturing experiences underlying material aesthetics. Among top-down approaches, rating methods are widely used [22, 30–32]. The semantic differential (SD; [33]), for instance, requires the individual to rate a specific entity regarding a given set of adjective pairs, with each pair consisting of a descriptor and its antonym. In the early 70s, building on the semantic differential method, Nagamachi introduced the concept of Kansei, which defines a “consumer’s psychological feeling and image regarding a new product” [34] (p. 4), and includes “using all the senses of sight, hearing, feeling, smell, taste as well as her cognition” [35] (p. 216). In the case of Kansei, the scales contain a descriptor and its negation [36]. Analogous to both of these techniques, the conceptual structure of materials aesthetics could be measured more objectively [36] using the adjectives obtained in our previous study to span the range of semantic evaluations in the present replication. To investigate the role of products, we chose an exploratory approach using a between-subjects design. Specifically, it was examined whether the evaluations of the adjectives diverge when individuals are explicitly instructed to reflect on the adjectives with respect to an individual pre-associated product, as compared to individuals who were not so instructed.

In the following, the results for the entire, aggregated sample used for the replication analysis are reported, supplemented by the most important results of the between-subjects design used to investigate the role of products.

## Materials and methods

### Participants

A final sample of 272 students (115 women, 156 men; 1 missing value), majoring in psychology (*n* = 141), educational science (*n* = 60), or “other” (*n* = 70), volunteered to participate in this study (1 missing value). The participants’ mean reported age was 23.7 years (*SD* = 4.4, ranging from 18 to 58 years; 3 missing values). Most reported German as their only native language (*n* = 251; 1 missing value). The majority did not consider themselves experts in the field of materials (*n* = 257; 1 missing value).

Participants were randomly assigned to the material categories (*n*_materials_ = 30; *n*_ceramics_ = 27; *n*_glass_ = 24; *n*_leather_ = 29; *n*_metal_ = 27; *n*_paper_ = 29; *n*_plastic_ = 20; *n*_stone_ = 28; *n*_textiles_ = 29; *n*_wood_ = 29) and to the conditions of the between-subjects design (S1 Text). The overall sample size was above the 177 participants needed, according to Cohen [37] (Table 3.4.1, p. 102) and the asymptotic relative efficiency (correction factor: 0.91; [38]), for the replication to detect a small effect of .02 with a correlation analysis (⍺ = 0.05; power = 0.80). Concerning the between-subjects design (*n*_no-product_ = 139, *n*_product_ = 133), an a priori power analysis showed that 63 participants per material category in each condition were required for detecting a large effect [37] using a multivariate analysis of variance (MANOVA; *f*^2^ = 0.35, ⍺ = 0.05; power = 0.80; G*Power 3; [39]). However, this number was not achieved in the present study (S1 Text). Nevertheless, the research question was investigated, as it aimed to examine potential differences based on the role of products in an exploratory manner, and pragmatic reasons of availability did not allow a greater sample size.

Three participants were not included in the final sample due to nonserious respondent behavior, that is, a tendency towards extreme values and no reported demographic variables, or only reporting demographic variables. The study received ethics approval from the university where the research was conducted, and was performed in accordance with the declaration of Helsinki. All participants gave their written informed consent prior to data collection, which was carried out anonymously. If requested, individuals received course credit for participating.

### Rating scales and procedure

The study was conducted online. Participants in the product condition first associated a product with the material category to which they were assigned, and all participants rated the adjectives for their assigned category. The product associations were listed prior to reflection on the adjectives. The instruction, here exemplified for glass, read as follows: “With what product do you spontaneously associate the material glass?” The instructions were adapted according to the specific material category (in case of the category materials in general, the phrase *the material* was omitted) and presented only visually (for the German instructions, see S1 Appendix). All participants then continued with the ratings. The instructions, presented visually and adapted as above, read as follows: “Please characterize the material glass [when incorporated in your associated product]. Rate the material glass using the scales below by ticking the value that you think is most applicable. Make your decision spontaneously and please assess all scales, even if some may seem inapplicable.” For the German instructions, see S2 Appendix.

In our previous study, 101 and 51 adjectives were respectively mentioned by at least 5% and 10% of the participants for at least one of the 10 materials and provided a basis for most of our analyses. For pragmatic reasons of feasibility and to reduce variability caused by idiosyncratic uses, terms mentioned by at least 10% of the participants were used to compile the rating scales in the present study (S1 Table, Column 1). A hybrid technique combining scaling procedures by Osgood et al. [33] and Kansei Engineers [40], as cited in [36], was applied: Participants rated the applicability of the 51 adjectives on individual 7-point unipolar scales ranging from 1 (*does not fit at all*) to 7 (*fits extraordinarily well*; for German anchors, see S2 Appendix). Participants were able to skip the rating of individual adjectives, although they were politely asked to rate all of them. Item sequences and the left–right orientation of the scale anchors were randomized.

A hybrid technique was used because both of the scaling procedures have advantages and disadvantages [36, 41] and neither Osgood’s nor Nagamachi’s technique seemed adequate for the ratings in the present study. Osgood’s technique provides a reduced number of ratings in total; however, it displays antonyms as mutually exclusive. The Kansei Engineers’ technique facilitates understanding and reduces time spent on the task, yet it often results in a skewed distribution [36]. In our previous study, *smooth* was the most frequently listed term, while its opposite, *rough*, was also among the most frequent concepts. But materials also pass through various different production processes, and depending on their eventual use, they can be *smooth* as well as *rough* [33], or *smooth* as well as *not smooth* [34]. This potentially holds for the remaining listed terms as well. In addition, several colors were listed in the previous study. But it does not seem possible to determine a complementary concept for every color—for example, brown—and it is not clear whether these concepts would really depict antonyms.

Finally, participants in the product condition were then asked to rate the pleasantness of their assigned material as incorporated in their associated product on a 7-point rating scale ranging from 1 (*not all pleasant*) to 7 (*very pleasant*) (S1 Appendix). Demographic data, including gender, age, native language, course of study, and whether they considered themselves experts on materials, were subsequently collected from all participants. No time limit was set for any part of the study.

## Results

### Replication

**Fig 1:**
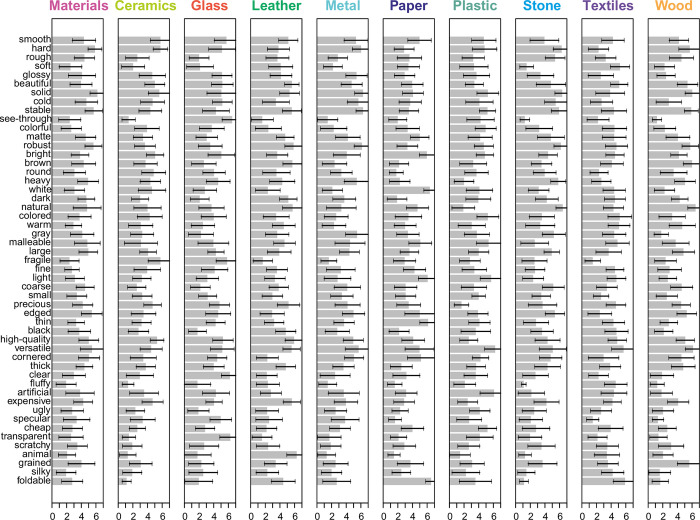
Means plotted for the 51 adjectives. Terms are ordered by overall relative frequency in the previous study. The error bars represent standard deviations.

The data from the present study were aggregated across participants, yielding overall mean scores for each adjective across the material categories (S1 Table, Column 3) as well as mean scores within each category (Fig 1). To test whether the present results replicated the previous ones, the relative frequencies (weighted by the sample size for the respective category; S1 Table, Column 2) obtained in the recent study were used for data analysis.

The overall mean scores correlated significantly with the overall relative frequencies, Spearman’s *ρ* = .50, *p* < .001, 95% CI [0.24, 0.69]; Kendall’s *τ* = .35, *p* < .001, 95% CI [0.17, 0.50]. Further, the means within individual material categories correlated significantly with the relative frequencies for the individual categories (Table 1; see S2 Table for all correlations). The lowest correlation was found for textiles and the largest for ceramics. *Versatile* (*M* = 5.43, *SD* = 1.65), *solid* (*M* = 5.41, *SD* = 1.63), and *stable* (*M* = 5.14, *SD* = 1.57) turned out to be the best-fitting adjectives. In the previous study, *solid* and *stable* were also among the 10 most frequent terms (14.2% and 9.3%, respectively), whereas *smooth* turned out to be the most central term for all target categories, followed by *hard* (45.1% and 26.7%, respectively). Both terms also had high means in the present study, respectively scoring sixth and fifth (*M* = 4.81, *SD* = 1.65; *M* = 4.83, *SD* = 1.91). *Fluffy* (*M* = 1.97, *SD* = 1.59), *silky* (*M* = 2.29, *SD* = 1.65), and *animal* (*M* = 2.31, *SD* = 1.99) were the worst fitting adjectives across all categories. In our previous study, the terms *silky* and *animal* were also among the least mentioned (1.4% and 1.9%, respectively). *Beautiful*, the primary and prototypical term for aesthetics, was rated with an overall applicability of *M* = 4.75 (*SD* = 1.55) and scored highest for leather and lowest for plastic (*M* = 5.62, *SD* = 0.98 and *M* = 3.40, *SD* = 1.23, respectively). Its opposite, *ugly*, was rated less applicable (*M* = 2.50, *SD* = 1.51) and scored highest for plastic and lowest for glass (*M* = 4.15, *SD* = 1.39 and *M* = 1.91, *SD* = 1.44, respectively). In the previous study, *beautiful* was mentioned with an overall frequency of 15.4%, being most frequently listed for materials in general and textiles (30.2% and 29.4%, respectively), and least frequently for plastic (3.9%). Its opposite, *ugly*, had an overall frequency of 2.9%, again being most frequent for textiles and materials in general (11.0% and 7.9%, respectively).

**Table 1:** Statistics of the correlation analyses between the two studies.

Category	Spearman’s *ρ*			Kendall’*s τ*		
	*ρ*	*p*	95% CI	*τ*	*p*	95% CI
Materials	.40	.004	[0.13, 0.62]	.29	.003	[0.11, 0.45]
Ceramics	.79	< .001	[0.63, 0.88]	.63	< .001	[0.50, 0.73]
Glass	.74	< .001	[0.56, 0.85]	.58	< .001	[0.44, 0.69]
Leather	.74	< .001	[0.56, 0.85]	.55	< .001	[0.40, 0.67]
Metal	.73	< .001	[0.54, 0.85]	.55	< .001	[0.40, 0.67]
Paper	.61	< .001	[0.38, 0.77]	.45	< .001	[0.29, 0.59]
Plastic	.73	< .001	[0.54, 0.85]	.56	< .001	[0.42, 0.68]
Stone	.71	< .001	[0.52, 0.83]	.53	< .001	[0.38, 0.65]
Textiles	.29	.03	[0.01, 0.53]	.21	.03	[0.02, 0.38]
Wood	.73	< .001	[0.54, 0.69]	.55	< .001	[0.40, 0.67]

The correlations are of the same material category between the present study and the previous study.

**Fig 2:**
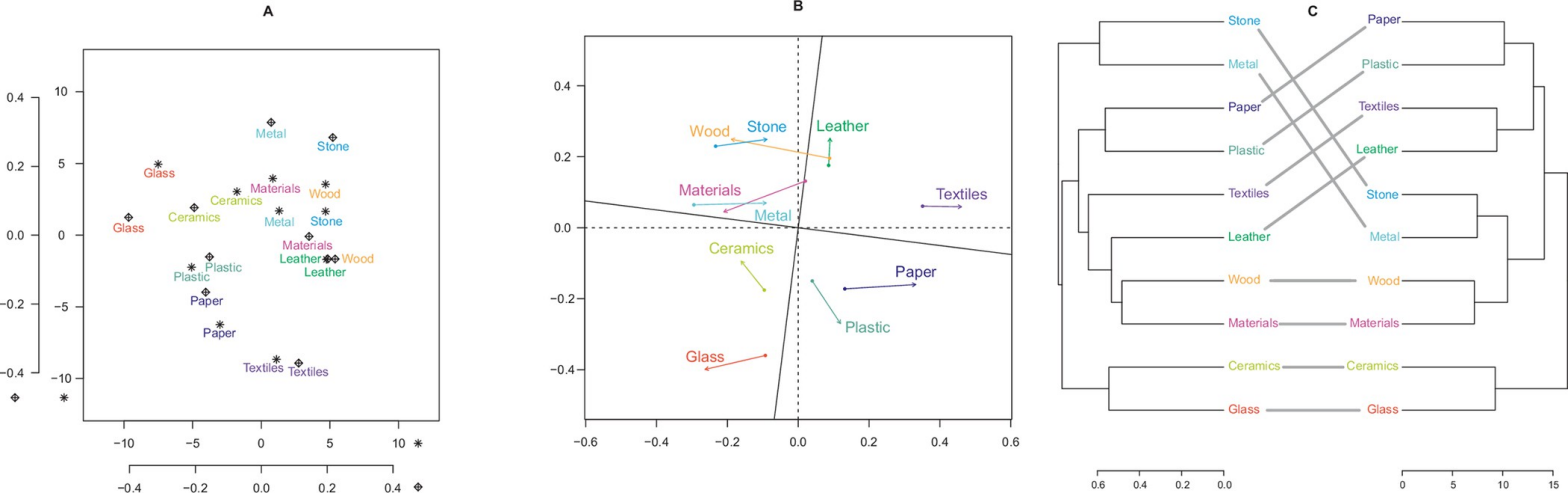
Visualizations of comparisons of the results obtained in the previous and the present study. A: The multidimensional scaling solutions of the two studies are overlaid in the same figure, with the present study represented by the stars. B: Procrustes analysis of the multidimensional scaling solutions of the two studies; errors are indicated by the colored arrows. C: Comparison of dendrograms for the two studies; the present study is depicted on the right.

Subsequently, dissimilarity matrices for the two studies were calculated. For the present study, Euclidean distances were calculated and for the previous one, Ružička similarity [42–45]. The matrices were each subjected to classical multidimensional scaling (MDS; [46]; two-dimensional; Fig 2A) and subsequently compared by Procrustes analysis [47, 48] (Fig 2B). This analysis yielded a significant correlation between the MDS results (sum of squares *m*^2^ = 0.32, *r* = .82, *p* = .001, 9,999 permutations). A Mantel correlation [49, 50] of the matrices also confirmed the similarity of the studies’ results (Mantel statistic *r* = .69, *p* = .001, 9,999 permutations). Further, the dissimilarity matrices were submitted to a hierarchical cluster analysis (HCA; complete linkage). Comparison of the two HCAs using the cophenetic correlation coefficient [51] yielded the similarity value .56. Visualizations of this analysis also mostly showed the similarity of the results, with entanglement of 0.22 (Fig 2C).

### Role of products

Overall, the results did not indicate that the conceptual structure of the aesthetics of materials varies as a function of specific products (S2 Text). Yet, some participants listed nonprototypical products. Therefore, a post hoc analysis considering only participants who listed prototypical products (*n* = 262) was conducted. To this end, individuals listing natural resources (i.e., *nature*, *water*), adjectives (i.e., *smooth*, *fragile*), material substances ([20]; *iron*, *steel*, *plastic*, *felt—*except for the category materials), and miscellaneous concepts (i.e., *with nothing*) as associated products were excluded (S3 Table, Column 4). The pleasantness rating for all products was *M* = 5.67 (*SD* = 1.33), and for prototypical products it was *M* = 5.71 (*SD* = 1.35).

MANOVAs comparing the means of the adjectives between the same material categories in the two conditions did not yield a significant effect in any of the cases (Table 2). The overall mean scores correlated significantly, Spearman’s *ρ* = .98, *p* < .001, 95% CI [0.96, 0.99]; Kendall’s *τ* = .89, *p* < .001, 95% CI [0.84, 0.92]. Regarding the 10 individual categories, the rank correlations between similar materials for both conditions were all significant, with the smallest correlation for plastic and the largest for leather and for wood (Table 2).

**Table 2:** Statistics of the post-hoc analysis in the between-subjects design.

Category	MANOVA							Correlation
	Wilk’s λ	*F*	*df*	Error *df*	*p*	*ηp2*	*ρ* (95% CI)	*τ* (95% CI)
Materials	.008	4.33	28	1	.37	0.99	.91* (0.83–0.95)	.77* (0.68–0.84)
Ceramics	.060	0.68	23	1	.76	0.94	.87* (0.76–0.93)	.71* (0.60–0.79)
Glass	.056	0.93	18	1	.69	0.94	.89* (0.80–0.94)	.72* (0.62–0.80)
Leather	.012	3.16	27	1	.42	0.99	.94* (0.88–0.97)	.80* (0.72–0.86)
Metal	.007	6.31	22	1	.99	0.99	.91* (0.83–0.95)	.76* (0.67–0.83)
Paper	.199	0.15	27	1	.99	0.80	.91* (0.83–0.95)	.77* (0.68–0.84)
Plastic	.004	15.48	17	1	.20	1.00	.80* (0.65–0.89)	.64* (0.51–0.74)
Stone	.121	0.28	26	1	.93	0.88	.93*(0.87–0.96)	.80* (0.72–0.86)
Textiles	.032	1.16	26	1	.64	0.97	.86* (0.75–0.93)	.70* (0.59–0.78)
Wood	.001	49.51	26	1	.11	1.00	.93* (0.87–0.96)	.81* (0.73–0.87)

*p < .001; the correlations are of the same material category between the two conditions.

Further, dissimilarity matrices for the two conditions (Euclidean distances) were calculated and each subjected to a classical MDS (two-dimensional) as well as an HCA (complete linkage). A Procrustes analysis of the two MDS solutions yielded a significant correlation (sum of squares *m*^2^ = 0.05, *r* = .97, *p* < .001, 9,999 permutations; S1 and S2 Figs). A Mantel correlation of the distance matrices also confirmed the similarity (Mantel statistic *r*: .83, *p* = .001, based on 9,999 permutations). Comparison of the two cluster dendrograms in terms of the cophenetic correlation yielded a similarity value of .61 (S3 Fig). Overall, the results of these analyses did not indicate a significant role of products.

## Discussion

The present study conceptually replicated our previous study [18] on the aesthetics of various materials. In addition, the potential role of products was examined in an exploratory manner. In the following, the results of both investigations are discussed individually.

### Replication

Overall, the present results support the findings obtained in our previous study. Specific outcomes provide a potential for discussion.

Frequencies and mean ratings, overall as well as within the individual material categories, correlated significantly between the two studies. Ceramics showed the greatest correlation, and textiles and materials in general the smallest. These three material categories differ in their versatility regarding their appearance and their use in and for products, for example, their color, shape, texture, and application.

Ceramics often have functional value and are used in limited contexts, for example, as bathroom fittings or kitchen utensils (S3 Table). Like mentioned previously, some participants in our previous study might have reflected on particular products rather than on materials per se. Even though instructed differently, this might be applicable to the participants in the no-product condition of the present study as well. Consequently, due to the limited versatility of ceramics, overlapping product associations between all participants in this category are conceivable.

Articles of clothing, however, are possible instruments for following the latest fashion and fashion trends, potentially leading to an ideal of beauty [6]. In the previous study, besides *beautiful*, textiles were frequently associated with the primarily haptic qualities *rough*, *soft*, *fluffy*, and *scratchy*. In the present study, *foldable*, *versatile*, *soft*, and *colored* were rated as most applicable. As indicated by the term *versatile*, textiles constitute a material with multiple uses, for instance, clothing, furniture, and car interiors. This interpretation is supported by the term *colored*. Color, a prevalent visual aspect of our environment [52], has been suggested to be the “most efficient dimension of discrimination” [53] (p. 330), which is also relevant for materials [54]. It is an effective sensorial property for attributing meanings to materials and products [16], and evaluations of colors can also vary according to the levels of category formation, or internal representations [55]. The combination of the terms *versatile* and *colored* indicates that, in contrast to ceramics, textiles are a multifold material. This may explain the variety of listed associations in the product condition resulting in differences between the two studies.

The same holds for materials in general. Rather than depicting a specific material, this is a superordinate category and includes all of the other materials used in this study and more, so that participants were less constrained. Interestingly, however, wood was the most listed product for this category. In our previous study, the categories materials in general and wood showed the greatest similarity, which is supported by the dendrograms for each study (Fig 2C). Similar to textiles, wood is omnipresent in everyday life, especially in construction and interior design. In addition, wood has already received much attention in previous research, for instance, regarding the preference for certain visual properties [56, 57], differences in perception of its properties based on the sensorial modality [58, 59] or on the varying naturalness of surfaces [60], and the representation of its tactile sensation in the brain [61, 62]. Due to its natural and traditional character, wood is often associated with craftsmanship [4], and has ecological value and potential for individuality due to visual imperfections [63]. Moreover, research indicates that its use in living areas has a positive impact on emotional states and psychological health [64–67]. Overall, wood is not only of major importance in environmental psychology, but also of specific value within the domain of materials in general.

Visual analyses also revealed similarities between the two studies in terms of the spatial configuration and also clustering of the conceptual structure of materials (Fig 2). This holds especially for glass and ceramics, as well as for wood and materials. Similarly, paper and plastic as well as metal and stone are arranged pairwise in both studies, indicating support for our previous findings.

*Versatile* was the most applicable term across all categories in the present study, whereas *smooth*, which had the highest frequency in our previous study, scored sixth. Objects from everyday life can hold functional and social information [26]. Materials that are incorporated in these objects of natural and built environments hold various meanings. Ashby and Johnson [4] (p. 89) refer to material as an “actor” that “can assume many different personalities, depending on the role it is asked to play.” As already mentioned above, analogous to the product experience [3], materials can differently gratify the senses, evoke meanings, and elicit emotions [15]. That is, depending on a material’s versatile appearance, it can be classified into multiple meaningful categories and elicit a variety of emotions. As Karana [15] (p. 62) put it, meanings of materials have a “versatile and dynamic character.”

In both studies, the term *beautiful* seemed to be of greater importance than its opposite *ugly*. Unsurprisingly, *ugly* was prevalent for plastic in both cases. This might indicate pro-environmental attitudes of the participating individuals. In addition, it is of great interest regarding the major issue of climate crisis and harmful consequences of the “throwaway society.” Environmental awareness is associated with the choice of specific materials [68]. As the Materials Experience Lab [69] summarizes: “One of the challenges of this century is to transform our current economy into an eco-friendly and self-sustaining system.” This *green aesthetics* [70] seems to be relevant not only for materials experts [71], but also for non-experts. In this context, plastics may have a rather negative reputation, as their recycling process is laborious [72]. In sum, it seems contradictory to ascribe plastics a positive aesthetic value such as beauty.

### Role of products

In an exploratory approach, the role of products in the conceptual structure of materials aesthetics was investigated using a between-subjects design. The results indicate that the conceptual system does not vary significantly as a function of specific products that incorporate the materials. Three possible reasons can be put forward, which should be investigated in future research: First, the conceptual structure of the domain in question might simply be independent of the material’s eventual use; second, the experimental approach may not have been effective for detecting underlying differences; and third, as indicated by the power analyses, the number of participants may have been too small to detect significant differences.

### Limitations

Participants were not obliged to evaluate each adjective if they chose not to. Yet, as Schütte and Eklund [36] have mentioned, individuals’ good compliance must be considered. That is, some might have rated adjectives, biasing their results, without having an opinion or properly understanding each adjective. This often results in a tendency toward ratings at the center, which in the present data, however, could not be observed.

Further, the present sample consisted of university students only, whereas for our previous study we additionally recruited individuals in the waiting rooms of citizen centers and vehicle registration authorities. However, the results showed great similarity between the student sample and the additional participants.

Based on the analyses of the between-subjects design, products do not play a significant role in the conceptual structure of the aesthetics of materials. Specific products associated with the different materials were collected using an exploratory approach that gave no restrictions. This method led to a great variety of products listed within each category in the product condition (S3 Table). However, a post hoc analysis investigating only prototypical products also did not reveal significant differences. Finally, it cannot be ruled out that some participants in the no-product condition also reflected upon particular products rather than materials per se. This, in turn, would imply that both conditions of the between-subjects design mapped identical content.

### Future research

Both the present and the previous study investigated the conceptual structure of materials aesthetics with reference to verbal expressions used in the German language and education system. In a recent study [73], for example, we investigated whether these long-term memory representations are influenced by specific expertise in the domain of materials. The studied group consisted of architects, designers, and interior designers. The results suggested that expertise modulates the conceptual structure of the aesthetics of materials to some degree and that it particularly influences the use of aesthetically evaluative terms. The concept of beauty turned to be even less relevant for this group than for the non-experts’ group [18]. Therefore, comparisons of and conceptual replications with different cultures, varying languages or degrees of (environmental) education would be instructive [74].

Individuals and objects are never part of only one category, but of several [26]. Similarly, it can also be assumed that each material belongs to multiple categories. Plastic, for instance, can belong to products initially associated with “modern housewives and the modern kitchen” [75] (p. 36), to the category of “cheap or trashy” materials [76] (p. 343), to the large contributors to CO_2_ emissions that have a large carbon footprint [77], and many more. Even though the findings are inconsistent [78], some authors suggest that the accessibility of attitudes towards (social) categories should be discussed to determine their applicability [79]. Attitudes, for example, Environmental Attitudes (EAs), were not assessed in the present report. EAs are understood as a “psychological tendency expressed by evaluating the natural environment with some degree of favor or disfavor” [80] (p. 1) and display “*concern* for the environment or caring about environmental issues (sometimes referred to as *pro-environmental attitudes*”; [81], pp. 65–66). It can be speculated, however, that EA biases the retrieval of the category to which a material belongs, for example, plastic as an environmentally harmful material, but also influences the conceptual structure of its aesthetics. To this end, a psychometrically sound inventory [82] could be used in future research (for a review of measuring pro-environmental behavior, see, e.g., [83]).

Place attachment and attachment to possessions would also be interesting to integrate into this line of research. Both have been studied intensively in the past [84–88]. The bonding between individuals and places or physical objects contributes to self-definition [89, 90]. As both places and physical objects incorporate materials, attachments may contribute to the conceptual structure of their aesthetics as well. Individuals attached to (near-)natural, rural places or organic objects, for instance, may have a different conceptual structure of certain materials, such as wood, than individuals attached to industrial, urban places or inorganic objects.

In this context, another aspect that was not controlled for but is also worthwhile to consider is the influence of culturally relative factors. The choice of specific building materials, for example, conveys information of the “creative expression, interpersonal style, and social class” of homeowners [27] (p. 155). Whether, for example, socio-economic backgrounds of the participants have an influence on the conceptual structure of the aesthetics of materials beyond house construction could be investigated.

Further research to elucidate the role of products in the conceptual structure of materials aesthetics appears to be worthwhile. Top-down approaches, for example, restrictions on product types to be listed and judged, could be interesting. In addition, as the pleasantness ratings of listed products indicated a positivity bias, future research might specifically focus on negatively valued entities.

## Conclusions

In sum, this study replicated previous findings on the conceptual structure of the aesthetics of various materials. In particular, the present results strengthen the outcome of the conceptual structure of the domain in question showing a primacy of sensorial, neutrally valenced, descriptive terms and highlight their versatile character. Moreover, as the concept of beauty does not have primacy in this domain, the conceptual structure of materials aesthetics differs from the conceptual representations underlying most other aesthetic domains. In addition, no significant influence of products into the conceptual representation of the aesthetics of materials was identified. Based on these insights, this study contributes to the corpus of existing studies on materials aesthetics as well as aesthetics in general.

## Supporting information

S1 Appendix Original German instructions for product association, pleasantness rating, and scale anchors in the product condition. (PDF)

S2 Appendix Original German instructions for rating scales and scale anchors in the product and no-product condition. (PDF)

S1 Text Descriptive statistics of the between-subjects design. (PDF)

S2 Text Results of the between-subjects design. (PDF)

S1 Fig Post hoc multidimensional scaling for prototypical products only. The multidimensional scaling solution of the no-product condition (A) and the product condition (B).
(PDF)

S2 Fig Post hoc Procrustes analysis of prototypical products only. Procrustes analysis of the multidimensional scaling solutions of the between-subjects design of the present study with prototypical products; errors are indicated by the colored arrows.
(PDF)

S3 Fig Post hoc comparison of dendrograms for prototypical products only. Comparison of dendrograms of the no-product condition (left) and the product condition (right) of the present study.
(PDF)

S1 Table Terms used in the rating scales and their statistics. English translation, original German adjective (in parentheses), percentage of occurrence with respect to sample size in Marschallek et al. (2021), and mean rating and standard deviation in the present study across all categories, with anchors 1 = *does not fit at all* to 7 = *fits extraordinarily well*.
(PDF)

S2 Table Correlations between identical material categories in the two studies. Rows indicate material categories in the present study. Columns indicate materials in Marschallek et al.’s (2021) study. *p < .05; **p < .01; ***p < .001.
(PDF)

S3 Table Associated products in the product condition. English translation of the associated products in the 10 material categories and the original German terms (in parentheses); absolute frequencies of their listing; and listings excluded in the post hoc analysis. ^a^ Products are ordered by frequency of occurrence. ^b^ Terms that were mentioned in both the singular and the plural are listed together in this table.
(PDF)
