# Supplementary material for: Materials aesthetics: A replication and extension study of the conceptual structure
Source: PLoS One. 2022 Nov 2;17(11):e0277082. doi: 10.1371/journal.pone.0277082 (PMC9629638; doi:10.1371/journal.pone.0277082)
Supplement: S1 Appendix — (PDF) [file pone.0277082.s001.pdf]

S1 Appendix. Original German instructions for product association, pleasantness rating, and scale anchors in the product condition.

Participants in the product condition received the following two instructions concerning the product association (1) and its pleasantness (2), adapted according to the specific material category and with the dots replaced by the corresponding German word (*Keramik*, *Glas*, *Stein*, *Leder*, *Metall*, *Papier*, *Kunststoff*, *Textilien*, *Holz*). In the case of the category materials in general (*Werkstoffe im Allgemeinen*), the phrase *the material* was omitted from the instructions:

(1) "Mit welchem Produkt assoziieren Sie spontan den Werkstoff … ?"

(2) "Wie angenehm finden Sie den Werkstoff …, wenn er für das von Ihnen zuvor genannte Produkt verwendet wird?"

The German anchors for the pleasantness rating were *überhaupt nicht angenehm* and *äußerst angenehm.*
