# Supplementary material for: Materials aesthetics: A replication and extension study of the conceptual structure
Source: PLoS One. 2022 Nov 2;17(11):e0277082. doi: 10.1371/journal.pone.0277082 (PMC9629638; doi:10.1371/journal.pone.0277082)
Supplement: S2 Appendix — (PDF) [file pone.0277082.s002.pdf]

S2 Appendix. Original German instructions for rating scales and scale anchors in the product and no-product condition.

The instructions were adapted according to the specific material category and the dots were replaced by the corresponding German word (*Keramik*, *Glas*, *Stein*, *Leder*, *Metall*, *Papier*, *Kunststoff*, *Textilien*, *Holz*). In the case of the category materials in general (*Werkstoffe im Allgemeinen*), the phrase *Werkstoffe im Allgemeinen* was omitted from the instructions. In the case of the no-product condition, the phrase *wenn er für das von Ihnen zuvor genannte Produkt verwendet wird* was omitted:

"Bitte charakterisieren Sie den Werkstoff … [wenn er für das von Ihnen zuvor genannte Produkt verwendet wird]. Schätzen Sie den Werkstoff … anhand der untenstehenden Skalen ein, indem Sie den Wert ankreuzen, der Ihrer Meinung nach am zutreffendsten ist. Treffen Sie Ihre Entscheidung spontan und beurteilen Sie bitte alle Skalen, auch wenn Ihnen eventuell einige unzutreffend erscheinen."

The German anchors for the rating scales were *trifft überhaupt nicht zu* and *trifft hervorragend zu.*
