# Supplementary material for: Materials aesthetics: A replication and extension study of the conceptual structure
Source: PLoS One. 2022 Nov 2;17(11):e0277082. doi: 10.1371/journal.pone.0277082 (PMC9629638; doi:10.1371/journal.pone.0277082)
Supplement: S1 Text — (PDF) [file pone.0277082.s003.pdf]

S1 Text. Descriptive statistics of the between-subjects design.

The no-product condition included 139 students (57 women, 82 men), majoring in Psychology (*n* = 73), Educational Science (*n* = 30) or “other” (*n* = 36). The participants’ mean reported age was 23.19 years (*SD* = 11.66, ranging from 19 years to 58 years; 1 missing value). Most of the participants (*n* = 128) reported German as their only native language. The majority of the participants did not consider themselves experts in the field of materials (*n* = 133). The number of final participants in each material category varied between 12 and 15 ($n_{materials}$ = 15; $n_{ceramics}$ = 13; n_{glass} = 13; $n_{leather}$ = 14; n_{metal} = 15; n_{paper} = 15; $n_{plastic}$ = 12; n_{stone} = 13; $n_{textiles}$ = 15; n_{wood} = 14).

The product condition included 133 students (58 women, 74 men; 1 missing value), majoring in Psychology (*n* = 68), Educational Science (*n* = 30) or “other” (*n* = 34; 1 missing value). The participants’ mean reported age was 21.46 years (*SD* = 15.31, ranging from 18 years to 36 years; 2 missing values). Most of the participants (*n* = 123) reported German as their only native language (1 missing value). The majority of the participants did not consider themselves experts in the field of materials (*n* = 124; 1 missing value). The number of final participants in each material category varied between 8 and 15 ($n_{materials}$ = 15; $n_{ceramics}$ = 14; n_{glass} = 11; $n_{leather}$ = 15; n_{metal} = 12; n_{paper} = 14; $n_{plastic}$ = 8; n_{stone} = 15; $n_{textiles}$ = 14; n_{wood} = 15).
