# Supplementary material for: Materials aesthetics: A replication and extension study of the conceptual structure
Source: PLoS One. 2022 Nov 2;17(11):e0277082. doi: 10.1371/journal.pone.0277082 (PMC9629638; doi:10.1371/journal.pone.0277082)
Supplement: S2 Text — (PDF) [file pone.0277082.s004.pdf]

S2 Text. Results of the between-subjects design.

In both conditions, the data were aggregated across participants in each material category, yielding overall mean scores for the adjectives in each condition as well as mean scores for the adjectives for each material category in each condition (see Figs 1 and 2 below). To test the similarity of the results obtained in each condition, we first conducted MANOVAs for each material category, comparing the means of the adjectives within the same category in the two conditions (see Table 1 below). Then we computed rank correlations between overall means and means within each category using Spearman's ρ and Kendall's τ. The overall mean scores in each condition correlated significantly, Spearman's $\rho = .98$, $p < .001$, Kendall's $\tau = .89$, $p < .001$. The mean scores for the same categories also correlated significantly (see Table S1). Next, the mean scores of the adjectives in each material category and condition were used to calculate dissimilarity matrices (Euclidean distances). These were then fed into classical multidimensional scaling (MDS) solutions (see Figs 3A and B below) and each subjected to a hierarchical cluster analysis (HCA) using complete linkage (see Figs 3C and D below). The two MDS solutions were then compared by Procrustes analysis (Jackson, 1995; Peres-Neto & Jackson, 2001). The analysis yielded a significant correlation between the MDS results (sum of squares $m^2 = 0.06$, $r = .97$, $p < .001$, 9,999 permutations; see Fig 4A below). A Mantel correlation (Mantel, 1967; Schneider & Borlund, 2007) of the Euclidean distance matrices also confirmed the similarity of the two conditions (Mantel statistic $r = .83$, $p = .001$, 9,999 permutations). Visualizations of the HCAs also indicated partial similarity of the results (see Fig 4B below; entanglement 0.25). Cophenetic correlation (Sokal & Rohlf, 1962) yielded a similarity value of .66.

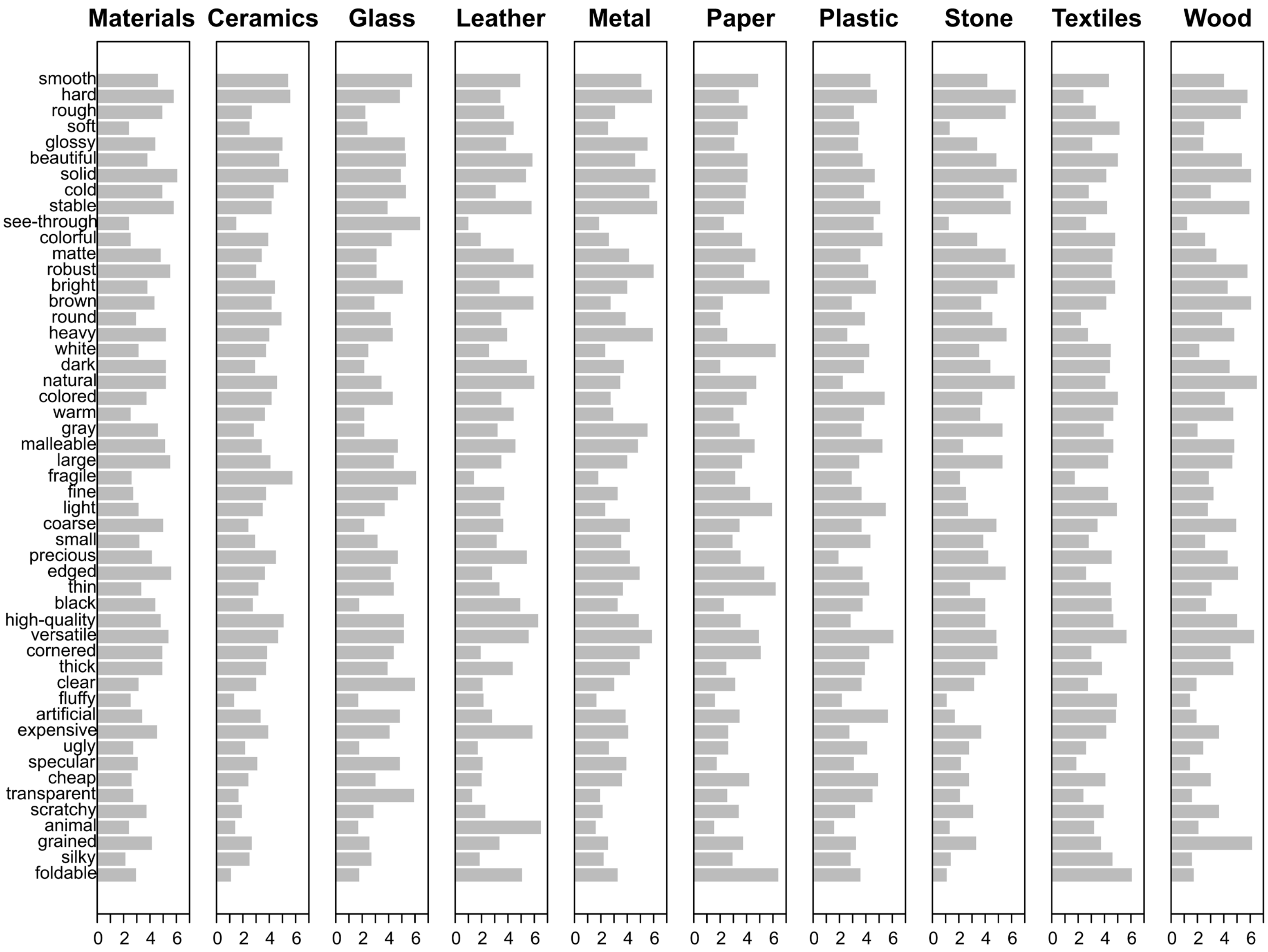
Materials
Ceramics
Glass
Leather
Metal
Paper
Plastic
Stone
Textiles
Wood
smooth
hard
rough
soft
glossy
beautiful
solid
cold
stable
see-through
colorful
matte
robust
bright
brown
round
heavy
white
dark
natural
colored
warm
gray
malleable
large
fragile
fine
light
coarse
small
precious
edged
thin
black
high-quality
versatile
cornered
thick
clear
fluffy
artificial
expensive
ugly
specular
cheap
transparent
scratchy
animal
grained
silky
foldable
0 2 4 6

Fig 1. Means plotted for the 51 adjectives in the present study's no-product condition, ordered by overall relative frequency in the sample of Marschallek et al. (2021).

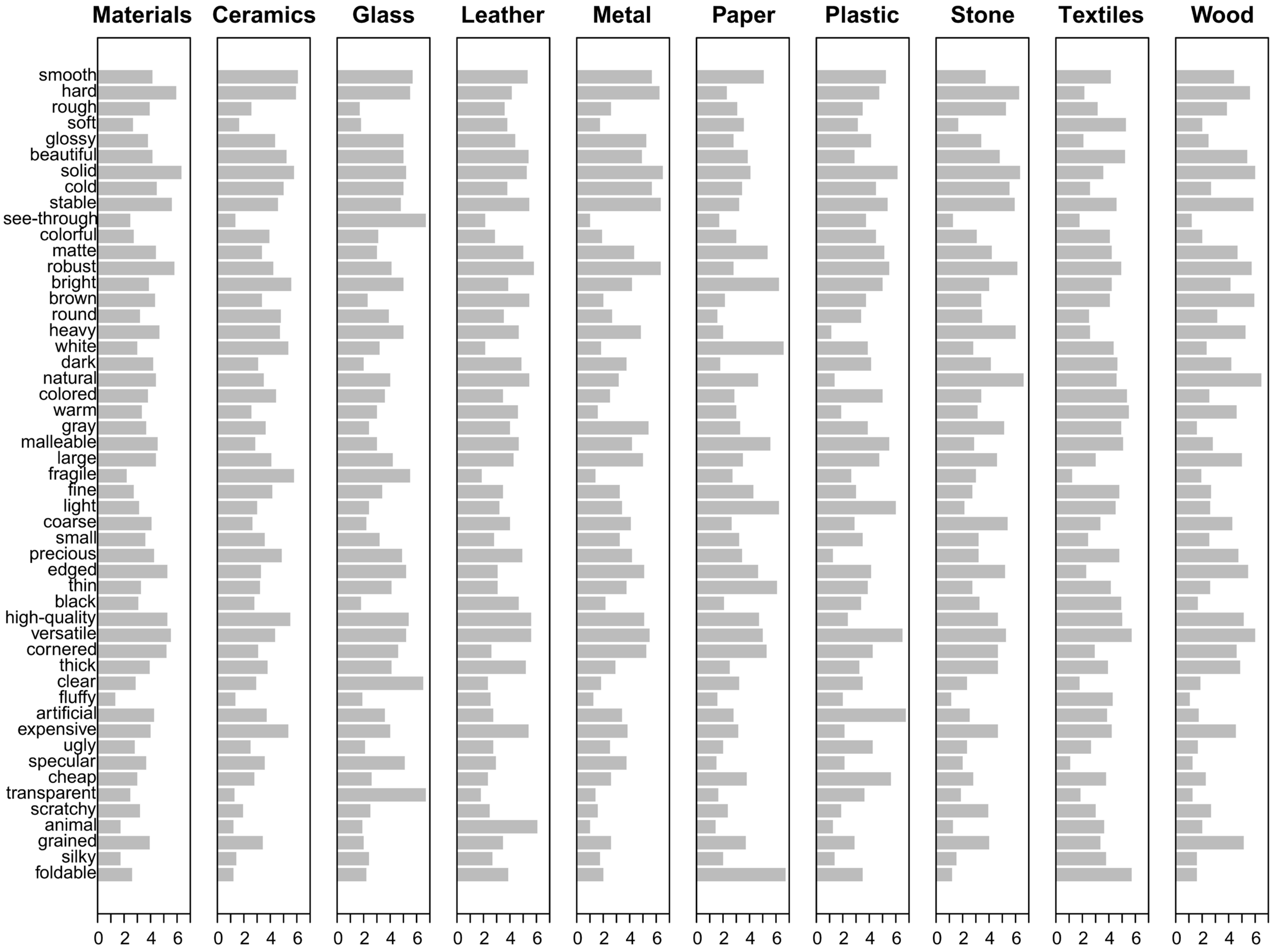
Materials
Ceramics
Glass
Leather
Metal
Paper
Plastic
Stone
Textiles
Wood
smooth
hard
rough
soft
glossy
beautiful
solid
cold
stable
see-through
colorful
matte
robust
bright
brown
round
heavy
white
dark
natural
colored
warm
gray
malleable
large
fragile
fine
light
coarse
small
precious
edged
thin
black
high-quality
versatile
cornered
thick
clear
fluffy
artificial
expensive
ugly
specular
cheap
transparent
scratchy
animal
grained
silky
foldable
0 2 4 6

Fig 2. Means plotted for the 51 adjectives in the present study's product condition, ordered by overall relative frequency in the sample of Marschallek et al. (2021).

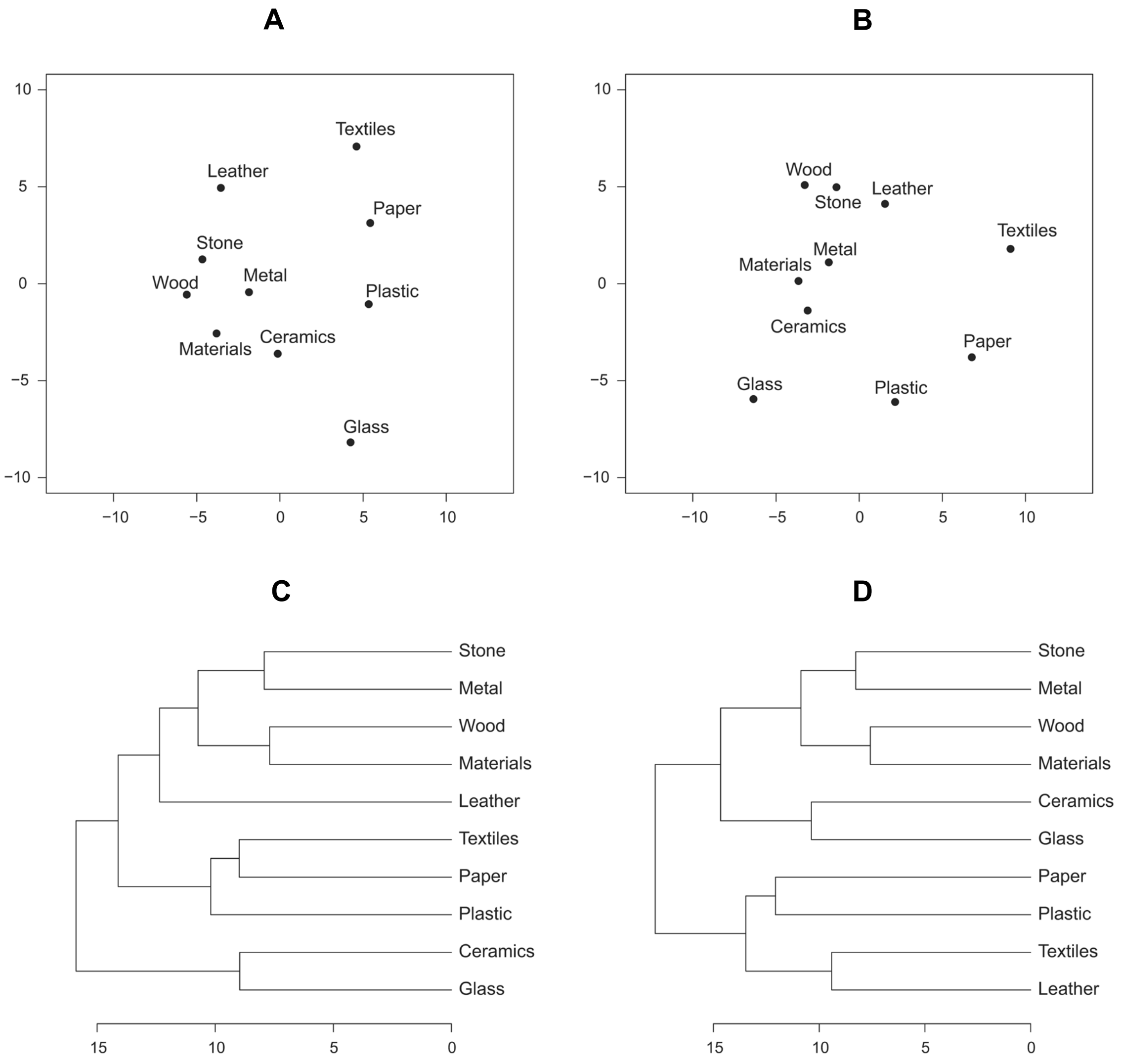

Fig 3. A: Two-dimensional multidimensional scaling solution for the ten categories in the no-product condition of the present study. B: Two-dimensional multidimensional scaling solution for the ten categories in the product condition of the present study. C: Dendrogram of

the hierarchical cluster analysis of the ten categories in the no-product condition of the present study. D: Dendrogram of the hierarchical cluster analysis of the ten categories in the product condition of the present study.

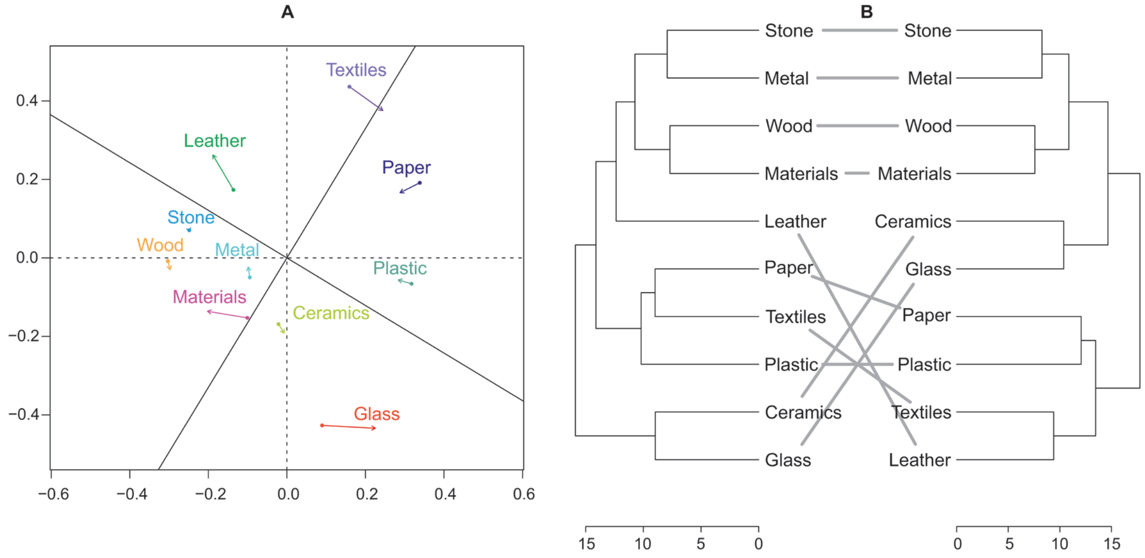

Fig 4. A: Procrustes analysis of the multidimensional scaling solutions of the between-subjects design of the present study; errors are indicated by the colored arrows. B: Comparison of dendrograms of the no-product condition (left) and the product condition (right) of the present study.

Table 1

Statistics of comparing identical material categories in the between-subjects design

Category	MANOVA				Correlation	
	Wilk's λ	*F*	*df*	Error *df*	*ρ*	*τ*
Materials	.008	4.33	28	1	.91***	.77***
Ceramics	.052	0.76	27	1	.88***	.72***
Glass	.077	0.57	21	1	.90***	.73***
Leather	.012	3.16	27	1	.94***	.80***
Metal	.048	0.79	25	1	.95***	.83***
Paper	.199	0.15	27	1	.91***	.77***
Plastic	.008	6.84	18	1	.81***	.64***
Stone	.121	0.28	26	1	.93***	.80***
Textiles	.017	2.14	27	1	.86***	.71***
Wood	.001	68.99	27	1	.95***	.83***

$^{***}p < .001$.
