# Supplementary material for: Materials aesthetics: A replication and extension study of the conceptual structure
Source: PLoS One. 2022 Nov 2;17(11):e0277082. doi: 10.1371/journal.pone.0277082 (PMC9629638; doi:10.1371/journal.pone.0277082)

S1 Figure. Post hoc two-dimensional multidimensional scaling for prototypical products only. The two-dimensional multidimensional scaling solution of the no-product condition (A) and the product condition (B).

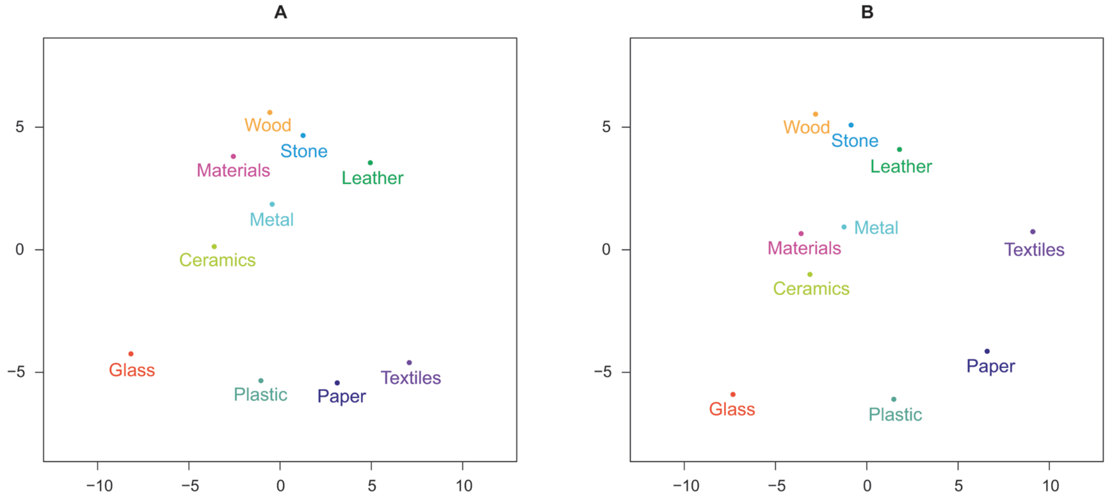

Supplement: S1 Fig — The multidimensional scaling solution of the no-product condition (A) and the product condition (B). (PDF) [file pone.0277082.s005.pdf]
