# Supplementary material for: Materials aesthetics: A replication and extension study of the conceptual structure
Source: PLoS One. 2022 Nov 2;17(11):e0277082. doi: 10.1371/journal.pone.0277082 (PMC9629638; doi:10.1371/journal.pone.0277082)

S2 Figure. Post hoc Procrustes analysis of prototypical products only. Procrustes analysis of the multidimensional scaling solutions of the between-subjects design of the present study with prototypical products; errors are indicated by the colored arrows.

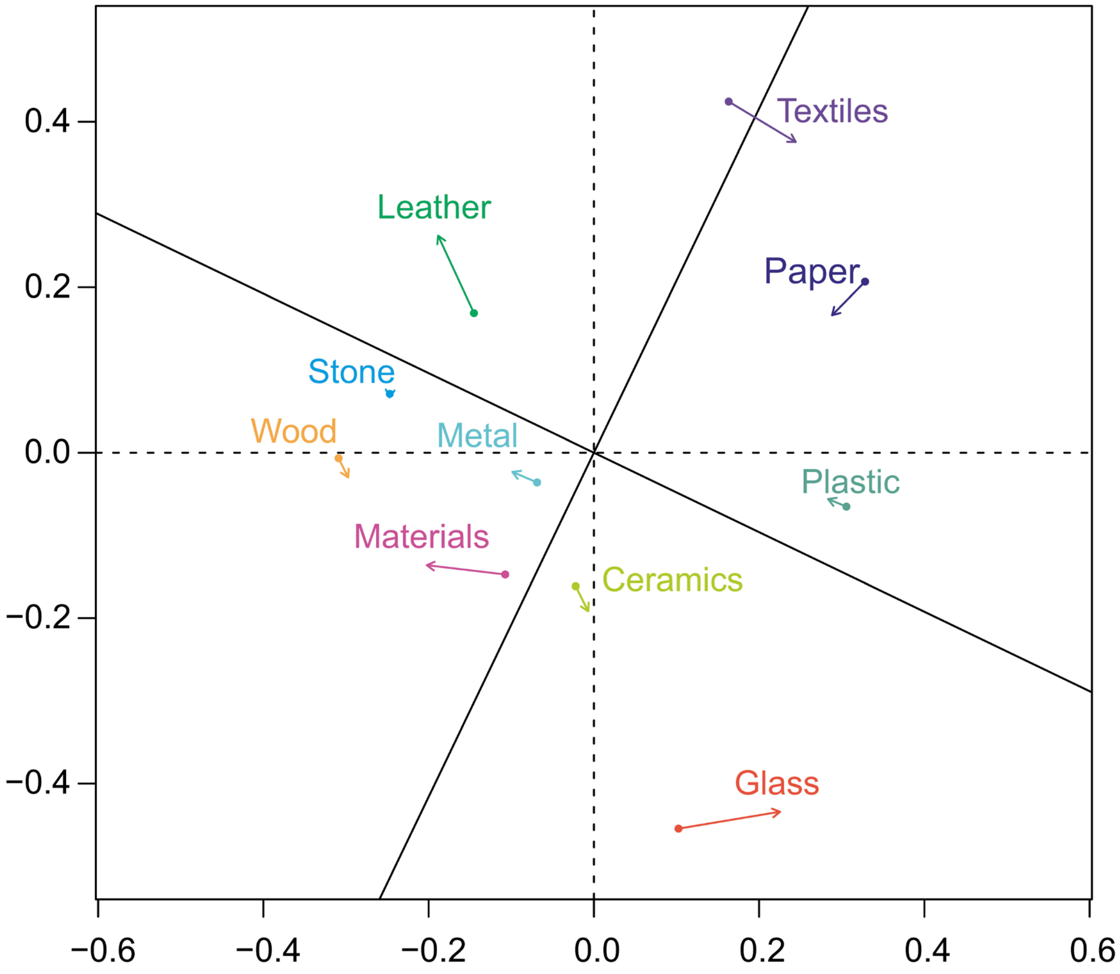

Supplement: S2 Fig — Procrustes analysis of the multidimensional scaling solutions of the between-subjects design of the present study with prototypical products; errors are indicated by the colored arrows. (PDF) [file pone.0277082.s006.pdf]
