# Supplementary material for: Materials aesthetics: A replication and extension study of the conceptual structure
Source: PLoS One. 2022 Nov 2;17(11):e0277082. doi: 10.1371/journal.pone.0277082 (PMC9629638; doi:10.1371/journal.pone.0277082)
Supplement: S3 Fig — Comparison of dendrograms of the no-product condition (left) and the product condition (right) of the present study. (PDF) [file pone.0277082.s007.pdf]

S3 Figure. Post hoc comparison of dendrograms for prototypical products only.

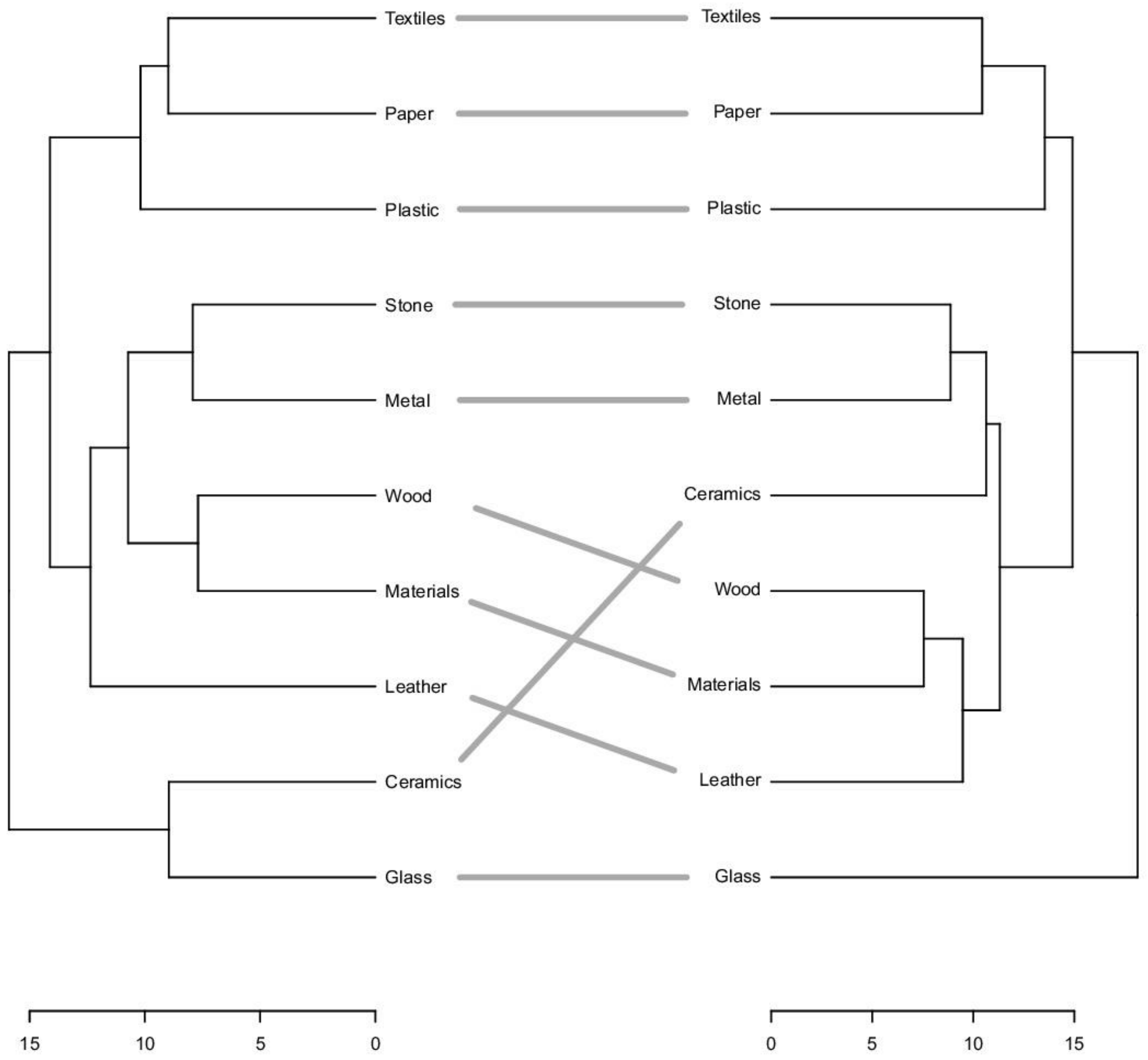

Note. Comparison of dendrograms of the no-product condition (left) and the product condition (right) of the present study.
