# Supplementary material for: Materials aesthetics: A replication and extension study of the conceptual structure
Source: PLoS One. 2022 Nov 2;17(11):e0277082. doi: 10.1371/journal.pone.0277082 (PMC9629638; doi:10.1371/journal.pone.0277082)
Supplement: S1 Table — English translation, original German adjective (in parentheses), percentage of occurrence with respect to sample size in Marschallek et al. (2021), and mean rating and standard deviation in the present study across all categories, with anchors 1 = does not fit at all to 7 = fits extraordinarily well. (PDF) [file pone.0277082.s008.pdf]

S1 Table. Terms used in the rating scales and their statistics.

Adjective	%	*M* (*SD*)
smooth (*glatt*)	45.1	4.81 (1.65)
hard (*hart*)	26.7	4.83 (1.91)
rough (*rau*)	26.4	3.61 (1.74)
soft (*weich*)	24.5	2.89 (1.83)
glossy (*glänzend*)	21.8	3.81 (1.85)
beautiful (*schön*)	15.4	4.75 (1.55)
solid (*fest*)	14.2	5.41 (1.63)
cold (*kalt*)	10.4	4.21 (1.88)
stable (*stabil*)	9.3	5.14 (1.57)
see-through (*durchsichtig*)	9.1	2.33 (1.98)
colorful (*bunt*)	8.7	3.27 (1.82)
matte (*matt*)	8.1	4.28 (1.59)
robust (*robust*)	7.6	4.99 (1.65)
bright (*hell*)	7.6	4.54 (1.49)
brown (*braun*)	7.6	3.83 (1.94)
round (*rund*)	7.3	3.38 (1.72)
heavy (*schwer*)	7.2	4.20 (1.90)
white (*weiß*)	6.8	3.52 (1.96)
dark (*dunkel*)	6.8	3.80 (1.69)
natural (*natürlich*)	6.7	4.65 (2.00)
colored (*farbig*)	6.6	3.85 (1.82)
warm (*warm*)	5.9	3.50 (1.83)
gray (*grau*)	5.9	3.76 (1.87)
malleable (*formbar*)	5.8	4.24 (1.98)
large (*groß*)	5.8	4.29 (1.43)
fragile (*zerbrechlich*)	5.7	2.88 (2.02)
fine (*fein*)	5.2	3.52 (1.64)
light (*leicht*)	5.1	3.69 (1.83)
coarse (*grob*)	4.9	3.73 (1.69)
small (*klein*)	4.7	3.18 (1.36)
precious (*edel*)	4.7	4.16 (1.68)
edged (*kantig*)	4.4	4.36 (1.88)
thin (*dünn*)	4.2	3.77 (1.64)
black (*schwarz*)	4.0	3.23 (1.78)
high-quality (*hochwertig*)	3.9	4.81 (1.54)
versatile (*vielseitig*)	3.8	5.43 (1.65)
cornered (*eckig*)	3.8	4.21 (1.85)
thick (*dick*)	3.7	4.00 (1.56)
clear (*klar*)	3.4	2.99 (1.96)
fluffy (*flauschig*)	3.0	1.97 (1.59)
artificial (*künstlich*)	3.0	3.48 (2.06)
expensive (*teuer*)	2.9	4.08 (1.58)
ugly (*hässlich*)	2.9	2.50 (1.51)
specular (*spiegelnd*)	2.7	2.68 (1.82)
cheap (*billig*)	2.6	3.17 (1.63)
transparent (*transparent*)	2.1	2.44 (1.95)

scratchy (*kratzig*)	2.0	2.83 (1.67)
animal (*tierisch*)	1.9	2.31 (1.99)
grained (*gemasert*)	1.9	3.55 (1.87)
silky (*seidig*)	1.4	2.29 (1.65)
foldable (*faltbar*)	1.1	3.23 (2.33)

English translation, original German adjective (in parentheses), percentage of occurrence with respect to sample size in Marschallek et al. (2021), and mean rating and standard deviation in the present study across all categories, with anchors 1 = *does not fit at all* to 7 = *fits extraordinarily well*.
