# Supplementary material for: Materials aesthetics: A replication and extension study of the conceptual structure
Source: PLoS One. 2022 Nov 2;17(11):e0277082. doi: 10.1371/journal.pone.0277082 (PMC9629638; doi:10.1371/journal.pone.0277082)
Supplement: S2 Table — Rows indicate material categories in the present study. Columns indicate materials in Marschallek et al.’s (2021) study. *p < .05; **p < .01; ***p < .001. (PDF) [file pone.0277082.s009.pdf]

S2 Table. Correlations between identical material categories in the two studies.

Category	1		2		3		4		5		6		7		8		9		10	
	ρ	τ	ρ	τ	ρ	τ	ρ	τ	ρ	τ	ρ	τ	ρ	τ	ρ	τ	ρ	τ	ρ	τ
1 Materials	.40**	.29**	.34*	.24*	.10	.07	.42**	.33**	.56***	.40***	.19	.14	.26	.18	.59***	.42***	-.07	-.05	.54***	.38***
2 Ceramics	.40**	.31**	.79***	.63***	.50***	.39***	.33*	.26**	.42**	.32**	.27	.19*	.23	.17	.47***	.34**	.04	.04	.40**	.29**
3 Glass	.11	.09	.42**	.32**	.74***	.58***	-.07	-.04	.23	.19	.22	.15	.29*	.20*	.11	.08	-.21	-.15	.02	.03
4 Leather	.25	.18	.29*	.20*	-.09	-.07	.74***	.55***	.35*	.28**	.12	.07	-.05	-.03	.29*	.19	.08	.05	.56***	.41***
5 Metal	.50***	.36***	.44**	.32**	.25	.19	.36*	.27**	.73***	.55***	.24	.16	.37**	.26**	.60***	.44***	-.02	-.02	.47**	.34**
6 Paper	.15	.10	.30*	.20*	.07	.05	.07	.05	.22	.15	.61***	.45***	.28*	.19	.18	.12	-.03	-.01	.26	.18
7 Plastic	.24	.16	.20	.16	.34*	.24*	.01	.00	.30*	.19	.42**	.29**	.73***	.56***	.22	.17	.01	.01	.06	.04
8 Stone	.50***	.37***	.38**	.27**	.13	.09	.41**	.31**	.55***	.41***	.22	.16	.17	.13	.71***	.53***	.06	.04	.56***	.42***
9 Textiles	.02	.02	.11	.09	-.27	-.18	.33*	.23*	.12	.08	.26	.18	.02	.02	-.08	-.07	.29*	.21*	.22	.15
10 Wood	.39**	.27**	.36**	.25*	.04	.02	.43**	.32**	.33*	.25*	.20	.12	.11	.08	.49***	.34**	.06	.04	.73***	.55***

Rows indicate material categories in the present study. Columns indicate materials in Marschallek et al.'s (2021) study. $^{*}p < .05$; $^{**}p < .01$; $^{***}p < .001$.
