# Supplementary material for: Materials aesthetics: A replication and extension study of the conceptual structure
Source: PLoS One. 2022 Nov 2;17(11):e0277082. doi: 10.1371/journal.pone.0277082 (PMC9629638; doi:10.1371/journal.pone.0277082)
Supplement: S3 Table — English translation of the associated products in the 10 material categories and the original German terms (in parentheses); absolute frequencies of their listing; and listings excluded in the post hoc analysis. a Products are ordered by frequency of occurrence. b Terms that were mentioned in both the singular and the plural are listed together in this table. (PDF) [file pone.0277082.s010.pdf]

S3 Table. Associated products in the product condition.

Category	All Products[ab]	*n*	Atypical Products
Materials	wood (*Holz*)	7	
	metal/s (*Metall/e*)	2	
	closet (*Schrank*)	1	
	house (*Haus*)	1	
	plastic (*Kunststoff*)	1	
	plastic (*Plastik*)	1	
	polystyrene (*Polystrol*)	1	
	ring (*Ring*)	1	
Ceramics	vase (*Vase*)	3	
	dishes (*Geschirr*)	2	
	brakes (*Bremsen*)	1	
	cups (*Cups*)	1	
	kitchen counter (*Küchenplatte*)	1	
	plate (*Teller*)	1	
	smooth (*glatt*)	1	smooth (*glatt*)
	teapot (*Teekanne*)	1	
	toilet bowl (*Kloschüssel*)	1	
	vases, cups, bowls (*Vasen, Tassen*, *Schüsseln*)	1	
	wash basin (*Waschbecken*)	1	
Glass	window (*Fenster*)	3	
	windowpane/s (*Fensterscheibe/n*)	2	
	balcony armchair (*Balkonsessel*)	1	
	bottle (*Flasche*)	1	
	fragile (*zerbrechlich*)	1	fragile (*zerbrechlich*)
	jars (*Einmachgläser*)	1	
	smooth (*glatt*)	1	smooth (*glatt*)
	water (*Wasser*)	1	water (*Wasser*)
Leather	shoes (*Schuhe*)	3	
	wallet (*Portemonnaie*)	3	
	belt (*Gürtel*)	2	
	jackets (*Jacken*)	2	
	bag (*Tasche*)	1	
	boots (*Stiefel*)	1	
	seats (*Sitze*)	1	
	gloves (*Handschuhe*)	1	
	pants (*Hosen*)	1	
Metal	a pipe (*ein Rohr*)	1	
	bicycle (*Fahrrad*)	1	
	car (*Auto*)	1	
	cutlery (*Besteck*)	1	
	gearwheel (*Zahnrad*)	1	
	hinge (*Scharnier*)	1	
	iron (*Eisen*)	1	iron (*Eisen*)
	knife (*Messer*)	1	
	steel (*Stahl*)	1	steel (*Stahl*)
	steel girder (*Stahlträger*)	1	
	steel plate (*Stahlblech*)	1	
	with nothing (*mit keinem*)	1	with nothing (*mit keinem*)
Paper	book (*Buch*)	4	
	spiral-bound notepad (*College Block*)	3	
	printer (*Drucker*)	2	

	DIN-A4 (*DIN-A4*)	1	
	DIN-A4 sheet (*DIN-A4 Blatt*)	1	
	kleenex (*Taschentuch*)	1	
	paper boards (*Kartons*)	1	
	toilet paper (*Toilettenpapier*)	1	
Plastic	air filter housing (*Luftfiltergehäuse*)	1	
	chair (*Stuhl*)	1	
	garbage bag (*Müllbeutel*)	1	
	garbage can (*Mülltone*)	1	
	Tupperware box(*Tupperdose*)	1	
	plastic (*Plastik*)	1	plastic (*Plastik*)
	plastic bags (*Plastiktüten*)	1	
	storage container (*Aufbewahrungsbehälter*)	1	
Stone	tile/s (*Fliese/n*)	3	
	house (*Haus*)	2	
	clinker (*Klinker*)	1	
	lamp (*Lampe*)	1	
	oven (*Ofen*)	1	
	paving slabs (*Gehwegplatten*)	1	
	pebble (*Kiesel*)	1	
	pickaxe (*Spitzhacke*)	1	
	stone axe (*Steinaxt*)	1	
	table (*Tisch*)	1	
	terrace (*Terrasse*)	1	
	tombstone (*Grabstein*)	1	
Textiles	clothing (*Kleidung*)	5	
	shirt (*T-Shirt*)	3	
	apparel (*Bekleidung*)	1	
	felt (*Filz*)	1	felt (*Filz*)
	garments (*Kleidungsstücke*)	1	
	hooded sweatshirt (*Kapuzenpullover*)	1	
	sewing machine (*Nähmaschine*)	1	
	sweater (*Pullover*)	1	
Wood	table (*Tisch)*	9	
	beam (*Balken*)	1	
	bench (*Bank*)	1	
	furniture (*Möbel*)	1	
	hammer (*Hammer*)	1	
	shed (*Schuppen*)	1	
	nature (*Natur*)	1	nature (*Natur*)

English translation of the associated products in the 10 material categories and the original German terms (in parentheses); absolute frequencies of their listing; and listings excluded in the post hoc analysis.

[a] Products are ordered by frequency of occurrence.

[b] Terms that were mentioned in both the singular and the plural are listed together in this table.
